# The Economic Performance of Reunited Families in Switzerland, 2013–2018

**DOI:** 10.1007/s12134-023-01047-3

**Published:** 2023-05-30

**Authors:** Juan Galeano, Roxane Gerber

**Affiliations:** 1 nccr – on the move, Neuchâtel, Switzerland; 2 Geneva School of Social Sciences, Institute for Demography and Socioeconomics (IDESO), University of Geneva, Geneva, Switzerland

**Keywords:** Immigration, Family reunion, Income, Integration, Switzerland

## Abstract

Family migration has gained prominence as one of the main reasons for international mobility in both Switzerland and the rest of western European countries. However, research aimed at evaluating the economic performance of reunited families has been constrained by the unavailability of individual income and/or household composition data. The joint use of population registers and information about individuals’ social security contributions has allowed us to overcome this limitation. Using transition matrices and logistic models, we assess the economic performance of reunited families at the household level and evaluate differences based on the region of birth of the person initiating the process, as well as the financial situation of these families 5 years after the reunion. The results show a process of economic convergence between the three groups under analysis despite the initial differences in the income level of families, and that most reunited families achieve satisfactory living conditions. They also highlight the hybrid nature of Swiss-headed reunited families, which initially resemble those headed by a non-EU/EFTA person, in terms of the contributions their members make to the household income, but after 5 years they contribute similarly to EU/EFTA headed reunited families.

## Introduction

Although family migration has declined during the last decade, it continues to be one of the most important reasons for international mobility in Switzerland and in many other European countries. According to the Federal Administration (SEM, 2020), 29% of immigrations to Switzerland in recent years were related to family reunion. However, there are notable sex differences among migrants; of surveyed migrants who arrived in Switzerland from 2006 to 2016, 40.5% of women declared family as one of the reasons for immigration while only 19.5% of men did (Wanner, 2019).

The importance of family migration has fuelled public and political debates about its convenience and the scope of family reunification rights in a country characterized by strict immigration laws. Aside from general discussions about whether family reunification favours or hinders integration in the medium or long run, the ultimate core of the debate concerns the economic productiveness of reunited migrants and their potential costs to the welfare state (Constant & Zimmermann, 2005; BASS, 2020). However, in a context of international competition to attract talent, policymakers are also aware that policies that facilitate family reunification attract and encourage the long-term settlement of skilled migrants (Khoo, 2014).

Although scholars have repeatedly studied the economic performance of the foreign population based on the reason for migration, most of these studies have approximated economic success from labour participation and/or employment rates due to the limited availability of income data (Cobb-Clark, 2000; Aydemir, 2011; Ruiz & Vargas-Silva, 2018) or from roughly aggregated data (Duleep & Regets, 1996). But perhaps more significant, even in those few cases where data was available (De Silva, 1997; Davidoff, 2006; Bevelander & Pendakur, 2014), the dilemma regarding the economic productiveness of reunited migrants has been tackled from an individual perspective consistent with the principles of the theory of human capital with very few exceptions (Cooke, 2003; Cooke et al., 2009; McKinnish, 2008). Although the quantification of the earning differential between reunited family members and labour migrants at the individual level contributes to inform policy design in this field, we consider that the debate about how reunited migrants fare economically would be enriched if framed into the broader context of family. This is the analytical perspective of this paper and the research gap we aim to fulfil. We evaluate, for the first time, in the Swiss case the economic performance or reunited families from a household perspective. For doing so, this research draws on a unique dataset combining the comprehensive register of immigrations from 2011 onwards, and the capability of reconstruct households, and follows them over time, with data on the annual professional income of individuals working in Switzerland. Its main objective is to assess the economic performance of reunited families and to evaluate differences among them based on the region of birth of the person initiating the process and the type of reunion.

## The Selectiveness of the Reunification Process: Institutional Settings

One of the contributions of this paper to the corpus of research on family reunion consists of portraying the barely researched Swiss case. Switzerland is one of the European countries with a higher share of foreign population (25% of the total population in 2020). In June 1999, Switzerland signed the Bilateral Agreement on the Free Movement of Persons with the EU, which came into force on 1 June 2002. Since then, EU/EFTA citizens are allowed to enter, live and work in the country with no special permission. On the other hand, Switzerland maintains a restrictive admission criterion based on quotas for highly qualified applicants from non-EU/EFTA countries. Regarding family reunion, citizens with a resident permit in Switzerland are allowed to bring in family members. However, conditions shift drastically depending on their nationality. EU/EFTA citizens, spouses/partners, descendants (children and grandchildren), ascendants (parents and grandparents), spouses/partners’ descendants and ascendants can be reunited as long as their financial independence from social assistance is guaranteed. In contrast, for non-EU/EFTA citizens, if the primary migrant is not married, a stable relationship needs to be proven as a condition (SEM, 2021) to enter the country, and only children under 18 years of age, of migrants with residence authorization, can be reunited. Lastly, for Swiss nationals, the current legal framework creates an unequal treatment for bringing some of their non-Swiss family members. In comparison to EU citizens, Swiss nationals face higher restrictions such a time limit for reunification and a narrower eligible family circle (ODAE-Suisse, 2012). This imbalance led to the presentation of a parliamentary motion in 2019 that will be debated by the Swiss Council in the near future (SFSO, 2021).^1^

States wield the power to shape family migration flows through the design of migration policies. The laws that give substance to these policies are typified, the requirements that must be met by both the person requesting for reunification and the person(s) to be reunited (Kraler & Bonizzoni, 2010; Strik et al., 2013). In Switzerland, the basic requirements for family reunion are adequate accommodation^2^ and sufficient assets to ensure the family will not depend on social assistance (Foreign Nationals & Integration Act, 2005). Reunited family members are also required to prove they know the local language spoken at their place of residence or, at least, to register for a language support programme (SEM, 2021). For non-EU/EFTA citizens, the conditions for family reunification are the same for migrants holding a settlement permit (type C, which is granted after 5 years of legal residence in Switzerland). Those with a temporary residence permit (type B) require extra approval of their applications by cantonal authorities (Foreign Nationals & Integration Act, 2005). Differences in the immigration regime between citizens of EU/EFTA countries and third-country nationals shape each other’s reasons for migrating to Switzerland. According to the Migration and Mobility Survey (Nccr-onthemove.ch, 2022), while the proportion of migrants coming to Switzerland for professional reasons reaches 70% for nationals from Germany, Italy, Spain and some other eastern European countries, it is no more than 30% for most people from the Balkan countries, Latin America or West Africa, for whom family reunion (or formation) is the main reason for migrating.

## Gender, Origin and Family Composition

Although family reunion is recognized as a human right (IOM, 2017) and as a promoter of well-being, access to the right of family reunion is unequal since it is regulated within the framework of neoliberal policies that aim at selecting the population with the best chance of integrating (professionally) in the host country. Scholars have introduced the concept of ‘civic stratification’ in the family migration studies to signalize those mechanisms of exclusion or inclusion of family members in the migration process that lead to a hierarchical classification of people (Kofman, 2018; Kraler, 2010; Kraler & Bonizzoni, 2010; Schweitzer, 2015). And, according to Bonjour and Duyvendak (2018), these mechanisms are in place to limit the arrival of migrants ‘with poor prospects’, in other words, low-educated, likely to be unemployed and potentially dependent on social welfare. Thus, gender, class, ethnicity, nationality and residence permit are then crucial determinants of civic stratification in the process of family reunification (Kraler, 2010; Kraler & Bonizzoni, 2010).

In the literature, migrants arriving to a country for family reasons are often called *tied*^3^ migrants. In other words, the *head*, *anchor* or *primary* migrant holds a residence permit while the residence permit of secondary immigrants depends on kinship relation with him or her. Despite some exceptions, such as the case of Latin American migration to Spain (Escribano & Martínez-Buján, 2014), women are more often *tied* migrants than men, and they account for the vast majority of those moving for family reasons (Cooke, 2013; Man, 2004; Wanner, 2019; Webb, 2015). It has been also shown, in the case of the Netherlands (Bonjour & Duyvendak, 2018), that political discourse related to family and gender norms often depicts the ‘migrant with poor prospects’ as a vulnerable, unemancipated tied migrant woman. In Switzerland, although the Federal Act on Foreigners and Integration of 2008 does not define reasons for migration on the basis of gender division, this continues to be, at least implicitly the case, as it considers the family migrant as dependent from the primary one, thus perpetuating a gendered stereotype of migration.

Very few quantitative studies measure the effect of migration on earnings by applying a gender perspective. It has been shown that women’s income tends to decrease after a migratory episode, while the impact of migration on the relative income of a couple has been compared with that of having a child (Cooke, 2003; Cooke et al., 2009; McKinnish, 2008). Moving for family reasons has been frequently also associated with a lower integration into the labour market and as having negative effect over the occupational status and employment rates, especially for women (Boyle et al., 2001; Meares, 2010; Purkayastha, 2005). After family migration, women (even those who are in a higher occupation than their partner) often experience a downgrade in their employment status (Boyle et al., 1999; Shauman & Noonan, 2007). In contrast to highly skilled economic migrants, who benefit from intercompany transfers and from an extended professional network that provides useful information in the host labour market, migrants who move for family reasons have a narrower professional network and need to rebuild it to enter the host labour market (Purkayastha, 2005). In the Swiss case, the lack of recognition of diplomas obtained in non-EU/EFTA countries is behind a higher likelihood of being overqualified when migration is motivated by family reasons (Pecoraro & Wanner, 2019; Wanner, 2019). And again, this is particularly the case for women, who are more prone to be excluded from the labour market, even if they are highly educated and were working before migrating (Gerber & Wanner, 2019). These cumulative disadvantages explain the major part of earnings losses for women after family migration.

But aside from structural constraints, gender norms related to work are also an important determinant of women participation in the labour market. For example, it has been shown that religious immigrant women participate less in the labour market and work fewer hours than nonreligious immigrant women and that for non-EUEFTA immigrant women, traditional gender-role attitudes partly explain their lower participation in the labour market in the destination country (Kanas & Müller, 2021). Migrant women’s lower participation into the host labour market can also be explained by the presence of children in the household, as women are often the main care providers within a family. In this sense, Switzerland is characterized by a shortage in family and institutional support for childcare as well as for the high cost of the private system (Adema et al., 2014; Felfe et al., 2013). The presence of children in the household has proven to engender direct and indirect costs that often deteriorate the financial situation of the family, which is particularly true for migrant families since they have fewer informal care-support networks than natives (SFSO, 2009). In the case of family reunion, the presence of children in the household may result from both the pre-existence of these children and the particular point in their family formation life course in which reunited partners find themselves. Thus, it is likely that the reunion of solely a partner (childless-reunions) would be followed by births during the first months or years in the destination country, limiting the access to the labour market for those women due to childcare obligations that are generally assumed by them.

Following the literature review, the two main hypotheses we aim to test are as follows.

Since labour market integration is more difficult for women (who are a majority of secondary migrants), and especially for non-EU/EFTA migrants for the above-mentioned reasons, we expect their contribution to the family budget to be lower than that of EU/EFTA migrants (H1).

Considering the lack of institutional support for childcare and the high cost of the private system in Switzerland, we expect that, the year following the reunion, the contribution of family members in reunions with children will be less than those in which the partner arrives alone (H2).

## Data and Methods

For this article, we rely on a unique dataset composed of two sources providing complementary information necessary for this research: Population Registers and Individual Income Information. On the one hand, the population registers record the basic sociodemographic characteristics of the population legally living in Switzerland. They also allow us to identify family reunions, reconstruct households and track them over time. The other information comes for the Old Age and Survivors Insurance (OASI)^4^ and allows us to accurately approximate the individual income of each member of a household. Within the OASI scheme, premiums are paid during work life and deducted from the salary received. In the case of unemployment, the premium is calculated on the contribution received by the unemployed person. These two records are part of the Swiss Longitudinal Database (Steiner & Wanner, 2015).

The process of creating our dataset can be summarized as follows: Our target populations are those individuals who were living alone on 31 December 2012 and in a situation of family reunification on 31 December 2013. Thus, it is important to recognize that we observe a selected group of family reunion migrants that move under a ‘lead’ and ‘follower’ dynamic. To identify this population, we first extract the permanent population aged 18 to 64 living alone in 2012. Then, we look for this population in 2013. We drop the cases of people living alone in 2012 and 2013 and of those living alone in 2012 and in households of 6 or more people in 2013. This last step, which sets the maximum size of households analysed in 2013 at 5, has two objectives. On the one hand, to exclude from the sample those families who moved to institutional households, but also to facilitate the process of rebuilding households by establishing the kin relation among its members. The population register records whether a person has entered Switzerland for family reunification reasons and his or her date of entry. However, among other limitations, it does not register the cases of couples in which both members (or at least the regrouped one) are citizens of an EU/EFTA country and have entered Switzerland declaring other reasons than family reunion. To include these cases in the sample, we use the information provided by the register on marital status, its date and the date of entry of family members. Although this procedure recovers a large number of cases, it does not collect those of common-law couples. Summarizing, our initial sample is composed of 7123 individuals defined as ‘heads’ (individuals who were in Switzerland in 2012) and 10,376 reunited family members in 2013. We divide this population according to three different places of birth of the head, i.e. Switzerland, EU/EFTA and non-EU/EFTA, and define five different types of family reunion:

- **Type 1:** Alone^5^ in 2012, with a reunited foreign partner in 2013.
- **Type 2:** Alone in 2012, with a reunited foreign partner and 1 child in 2013.
- **Type 3:** Alone in 2012, with a reunited foreign partner and 2 children in 2013.
- **Type 4:** Alone in 2012, with a reunited foreign partner and 3 children in 2013.
- **Type 5:** Alone in 2012, with a reunited child and without a partner in 2013.

After an initial descriptive analysis of our dataset, we built a transition matrix to assess changes in the composition of households, and its size, in 2018. Once households are reconstructed, we track the contribution of all family members between 18 and 64 years old in 2014 and 2018 to the OASI fund to estimate the household’s income. Income thresholds are set in relation to the median equivalent income of heads in 2012 and of families in 2014 and 2018 following the proposal of the OECD and EUROSTAT considering the number of people in the household (OECD, 2017). Therefore, a factor of 1 is applied for family heads, 0.5 for family members over 14 years old and 0.3 for family members below 14 years old. We classify households as ‘very low-income (VLI)’ households when they earn less than 50% of the median income in a given year. ‘Low-income (LI)’ households are those who earn between 50 and 60% of the median income and ‘median or high income (MHI)’ the rest of the cases. These thresholds are widely used in Swiss Federal Offices’ reports (see for instance Wanner & Gerber, 2022 for the Federal Social Insurance Office) and were firstly proposed by the OECD to measure relative poverty (OECD, 2017).

To assess the economic performance of households, first we compute a transition matrix between the income position of the heads in 2012 and their families in 2014. Second, we fit a logistic regression model to evaluate the probability of being below the median income after family reunion controlling by the income level of the head of the household in 2012, the type of reunion, the region of birth of the partner and the relative share of the contribution of family members to the household budget. In a last step, we reassess the income level of households in 2014 and 2018 focusing on how families in LI or VLI households in 2014 managed to overcome that situation by 2018.

## Results

### Results 1.1: Descriptive Analysis of the Dataset

As mentioned above, our initial population is composed of 7123 individuals classified as ‘heads’. Regarding their place of birth, 38% were born in an EU/EFTA country (Portuguese, French and Italians represent two out of three people in this group), 33% in a non-EU/EFTA country (119 nationalities, none of which exceeds 9% of the total of this group) and 29% in Switzerland (92% of them with Swiss nationality). Our dataset portrays family reunion in Switzerland as a male-driven process. Three out of four of the heads (78%) were men, a share that rises to 83% in the case of the EU/EFTA population. The degree of masculinization of those who initiate a reunion process also increases in relation to the type of reunion. In those reunions that involve a partner and children, the proportion of male heads surpasses 80% and illustrates one of the more common strategies of migrant families (Cooke, 2013; Man, 2004; Wanner, 2019; Webb, 2015).

The most frequent type of family reunion for the three population groups is the reunification of a partner, which represents two-thirds (66%) of total cases and rises to 77% in the case of the Swiss-born population (Table 1). The differences observed in relation to the relative distribution of the types of reunion by region of birth of the head can be explained both by the interplay of an age effect, different migratory strategies and a different set of motivations. For example, the higher proportion of reunions of a partner and two or three children by the EU/EFTA population in comparison to the non-EU/EFTA is connected to the younger age structure of the latter (40.1 vs 37.5 years), although it cannot be ruled out that some part of the difference could be also due to a deliberate migration strategy for some non-EU/EFTA-headed families. On the other hand, the different share of reunions of a partner without children between the EU/EFTA and the Swiss population (both with the same mean age) would illustrate the different set of motivations. The high prevalence of this type of reunion among the Swiss population is connected to the fact that family reunion and family formation usually overlap (Riaño, 2011). Our dataset confirms this feature; 93% of these couples got married the year of the reunion or the year before. In terms of the region of birth of the reunited partners, it should be noted that 83.5% of the partners, when the reunion was headed by a Swiss citizen, were nationals from non-EU/EFTA countries. In the case of the reunions headed by non-EU/EFTA migrants, 88% of partners were also from a non-EU/EFTA country and when the reunion was headed by an EU/EFTA migrant, 66% of partners were also from an EU/EFTA country.

**Table 1:** Distribution of heads by place of birth, sex and reunion type, 2013

	EU/EFTA			Non-EU/EFTA			Switzerland		
Reunion type	Male	Female	Total	Male	Female	Total	Male	Female	Total
Total	2250	452	2702	1809	538	2347	1501	573	2074
Partner	*54.5%*	*69.5%*	*57.0%*	*64.4%*	*79.0%*	*67.7%*	*74.6%*	*82.0%*	*76.7%*
Child alone	*3.5%*	*10.0%*	*4.6%*	*2.7%*	*5.2%*	*3.2%*	*2.1%*	*1.7%*	*2.0%*
Partner and 1 child	*23.4%*	*13.3%*	*21.7%*	*19.7%*	*11.2%*	*17.7%*	*15.5%*	*10.5%*	*14.1%*
Partner and 2 children	*14.8%*	*4.6%*	*13.1%*	*9.1%*	*3.5%*	*7.8%*	*5.5%*	*4.0%*	*5.1%*
Partner and 3 children	*3.8%*	*2.7%*	*3.6%*	*4.2%*	*1.1%*	*3.5%*	*2.3%*	*1.7%*	*2.1%*

Source: Own elaboration with data from the Population Registers

In 2013, our initial population (7123 heads) had reunited a total of 10,376 people. Five years later, we still find living in Switzerland 85% of this population. However, the survival rate of these reunited families varies considerably depending on the region of birth of the head of the household and it ranges between a maximum of 90% for the Swiss-headed families to 78.2% for those headed by an EU/EFTA national (Table 2). The lower survival rate of EU/EFTA-headed families can be explained considering the greater intra-European mobility of this group, but also as part of an economic optimization strategy for some families who, although they work in Switzerland, decide to live in the border areas of France, Germany or Italy due to lower cost of housing and life in general.

**Table 2:**
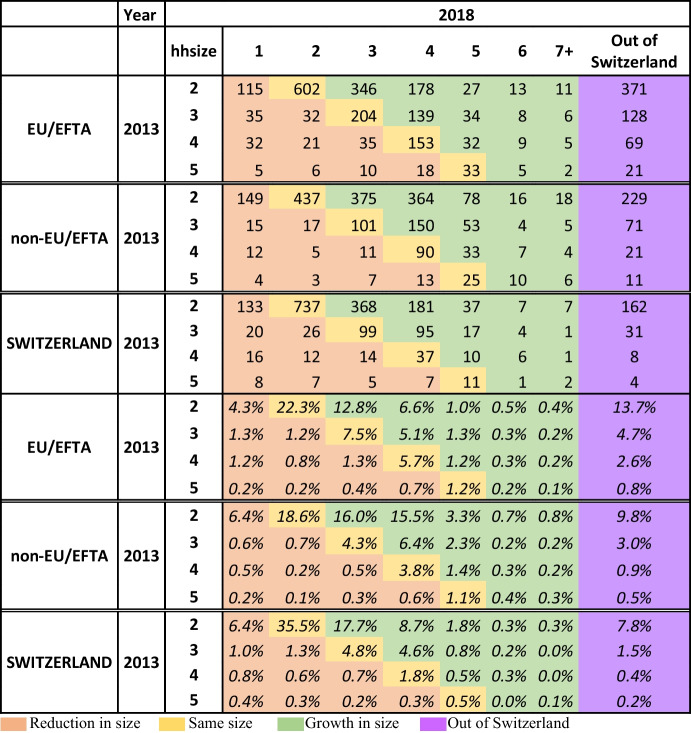
Transition matrix between household size in 2013 and 2018 by region of birth of the head

Source: Own elaboration with data from the Population Registers

The transition matrix between the size of the household in 2013 and 2018 also allows evaluating their evolution in terms of stability (yellow cells), growth (green cells) or decrease (orange cells) in the number of people they are made up of (Table 3). In this sense, while 42.6% of the households headed by a Swiss remain the same size 5 years after the reunion, the share decreases to 36.7% for those headed by an EU/EFTA national and to 28% for the non-EU/EFTA. The lower stability of the latter translates into a higher growth of them. Almost half of these households (48%, 1123 cases) increased their size between 2013 and 2018. When the change involves an increase in the number of people for any of the 3 groups, it is mainly related to the addition of new members to the family, either by birth or as a result of a new reunion. Thus, out of the 4833 new people we found in these households in 2018, two-thirds (66.4%) were 5 years old or less and 72% were under 19 years old. On the contrary, Table 2 also shows that one in ten households experienced a reduction on its size in 2018 with no significant differences between groups, and that the highest incidence occurred among two-person households. Although we do not have information on the actual reason that explains the reduction in household size, we can assume that it is related to the dissolution of the union and/or the emancipation of the children. But it could also be the case that it is a family decision on the return of the reunited members to the country of origin or their (re)emigration to another country, giving place to the constitution of transnational families.

**Table 3:** Distribution of families by region of birth of the head, type of reunion and income type of household in 2014

	EU/EFTA				Non-EU/EFTA				Switzerland			
	MIH	LIH	VLIH	Out of CH	MHI	LIH	VLIH	Out of CH	MHI	LIH	VLIH	Out of CH
Total	2022	209	358	116	1298	240	680	126	1540	115	366	53
Partner	*60.9%*	*36.8%*	*45.8%*	*57.8%*	*74.7%*	*60.0%*	*60.6%*	*51.6%*	*79.0%*	*75.7%*	*69.9%*	*58.5%*
Child alone	*4.2%*	*5.3%*	*6.1%*	*4.3%*	*2.5%*	*5.0%*	*3.8%*	*4.8%*	*1.6%*	*0.9%*	*3.8%*	*3.8%*
Partner and 1 child	*20.4%*	*31.1%*	*24.3%*	*18.1%*	*14.9%*	*20.8%*	*20.1%*	*27.8%*	*12.9%*	*18.3%*	*16.9%*	*20.8%*
Partner and 2 or 3 children	*14.4%*	*26.8%*	*23.7%*	*19.8%*	*7.9%*	*14.2%*	*15.4%*	*15.9%*	*6.5%*	*5.2%*	*9.3%*	*17.0%*

Source: Own elaboration with data from the Population Registers and the Old Age and Survivors Insurance

### Results 1.2: Family Reunion and Income the Year After the Reunion

The main goal of this paper is to evaluate the economic performance of reunited families. To do so, we assess the income level of heads in 2012 and of their households in 2014, the year after the reunion.

Although it has been commonly assumed that family reunion is associated with (economic, housing and labour) stability in the host country (Ambrosini, 2015; Castles & Miller, 2003; Kulu & Milewski, 2007), some studies have shown that is not always the case, and that some migrants reunify family members with the support of social and family networks despite being in an economically tight situation (González-Ferrer, 2011; Fresnoza-Flot, 2017; BASS, 2020). In 2012, 77.3% of heads (5502 cases) in our dataset were living in MHI unipersonal households (share that ranges between 72% for the non-EU/EFTA to 81% for the Swiss population). Therefore, before the reunion, 22.7% of heads were already below the median income, showing that even in a country with a highly restrictive immigration policy such as Switzerland, family reunion may occur despite the individual initiating the process being in a disadvantaged economic position.

The year after the reunion, in 2014, 2 out of 3 families (68.2%, 4860 cases) in our sample were living in MHI households, 27.6% in households below the median income and 4.1% were no longer in Switzerland (Table 3). As could be expected due to the limitations and selectivity imposed by the government to bring family members to Switzerland, a majority of reunified families achieve an income that allows them to live at least in median income households. However, this aggregate image conceals notable differences based on the region of birth of the head of the household and its size. For example, 40% of reunited families headed by a non-EU/EFTA migrant were living in LI or VLI households in 2014, while this share drops to 21% for EU/EFTA-headed reunions and to 23% when the reunion was headed by a Swiss national. As expected, the proportion of families in LI or VLI households is positively correlated with its size. The case of family reunions of a partner and two or three children rises to 54% among those households headed by a non-EU/EFTA person (31% of EU/EFTA and to 26.8% for Swiss-headed families).

**Fig. 1:**
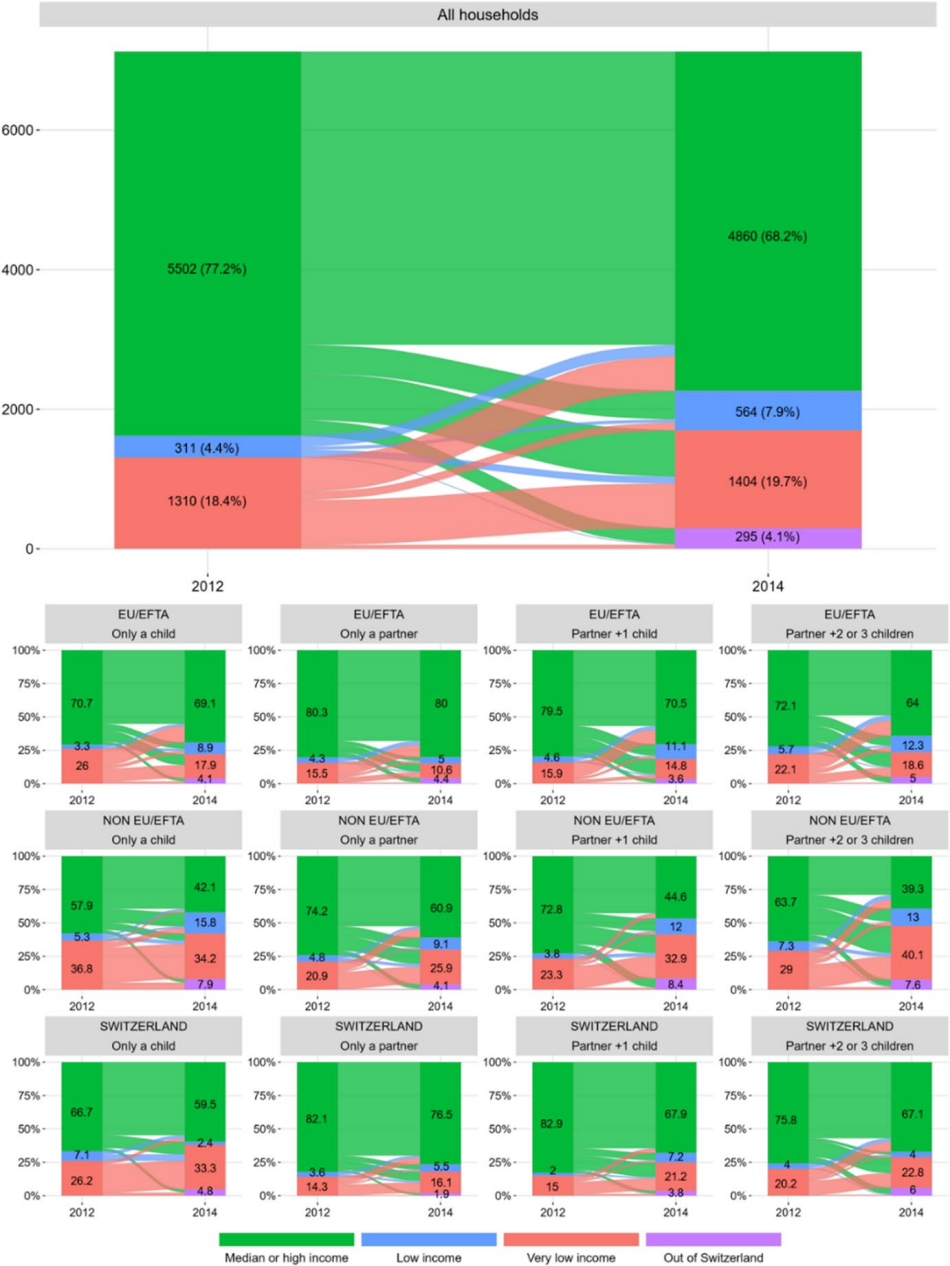
Transition between income states of heads in 2012 and households in 2014. Source: Own elaboration with data from the Population Registers and the Old Age and Survivors Insurance

However, does living in households below the median income in 2014 derive from the addition of family members to the household? Is it related to a ‘poor’ economic performance of the head of the family before the reunion? Or is it a mix of both factors? To answer these questions, we build a transition matrix comparing the income position of heads in 2012 and of reunited families in 2014. Figure 1 presents in a visual manner the transitions among the possible states between the 2 years. In the second set of panels, the results are disaggregated according to the region of birth of the head and the type of reunion. The transition matrix reveals that 45.4% of those families living in households with an income below the median in 2014 were headed by someone who was already in that situation back in 2012. Thus, in these cases, the disadvantaged situation of the household in 2014 seems to be mainly connected to the poor economic performance of the head of the household prior to the reunion. Surprisingly, the proportion of heads living on an income below the median in 2012 slightly increases with the number of reunited family members in 2013, what might seem a lack of foresight or even recklessness. However, the proportion of households that overcome this situation after the reunion also seems to be correlated with the number of reunited family members. This is particularly the case for Swiss- and non-EU/EFTA-headed families and reflects the strategic dimension that family reunification entails in economic terms for these households. For the remaining 54.6% of households living below the median income in 2014, this situation results, on the one hand, from the readjustment of the income threshold linked to the incorporation of new members to the household as also, in some cases, from the decrease in the income of the head between 2012 and 2014.

On the opposite direction, 4 out of 10 heads in LI and VLI households in 2012 overcame this situation after the reunion. This ‘economic boost’ effect of family reunion is especially accentuated among households headed by immigrants from EU/EFTA countries (58% of those who were below the median income in 2012), and it is less so for those non-EU/EFTA- or Swiss-headed reunions (around 27% of those who were below the median income in 2012). The reasons behind this improvement are of two types. On the one hand, the increase in the income of the head of household and, on the other, the contribution that the reunited members make to the family budget. Among households headed by an EU/EFTA national, the first of these reasons explains most of this improvement, while in those headed by a Swiss or a non-EU/EFTA national, the contribution of family members was crucial to improve the economic situation of the household (53% of the cases). This analysis also highlights the double disadvantage faced by non-EU/EFTA families. On the one hand, a worse initial position with a higher share of household heads’ living below the median income before the reunion and, on the other, a lower share of households overcoming that situation after it.

### Results 1.2.1: the Economic Contribution of Family Members

As seen in previous section, living above or below the median income after family reunion is not the exclusive outcome of the economic performance of the head of the household but it also depends on the number of relatives reunited and their contribution to the family income. However, and as shown by previous research (Davidoff, 2006), the contribution of family members to the household budget shortly after arrival tends to be scarce. In 2014, the year after the reunion, income data shows that for 47% of families the contribution of family members was nil (Table 4).

**Table 4:** Distribution of families by region of birth of the head, type of reunion and income contribution of family members in 2014

	EU/EFTA				Non-EU/EFTA				Switzerland			
	Zero	Below^1^	Above^2^	Total	Zero	Below	Above	Total	Zero	Below	Above	Total
Partner	582	306	547	1435	709	356	427	1492	642	410	459	1511
Child alone	99	13	4	116	63	5	2	70	35	3	0	38
Partner and 1 child	272	163	127	562	244	89	43	376	144	89	40	273
Partner and 2 or 3 children	188	139	104	431	124	77	31	232	71	34	30	135
Partner	*40.6%*	*21.3%*	*38.1%*	100%	*47.5%*	*23.9%*	*28.6%*	100%	*42.5%*	*27.1%*	*30.4%*	100%
Child alone	*85.3%*	*11.2%*	*3.4%*	100%	*90.0%*	*7.1%*	*2.9%*	100%	*92.1%*	*7.9%*	*0.0%*	100%
Partner and 1 child	*48.4%*	*29.0%*	*22.6%*	100%	*64.9%*	*23.7%*	*11.4%*	100%	*52.7%*	*32.6%*	*14.7%*	100%
Partner and 2 or 3 children	*43.6%*	*32.3%*	*24.1%*	100%	*53.4%*	*33.2%*	*13.4%*	100%	*52.6%*	*25.2%*	*22.2%*	100%

Source: Own elaboration with data from the Population Registers and the Old Age and Survivors Insurance

^1^Below: The contribution of the members of the household is less than the proportional income they should contribute to the household

^2^Above: The contribution of the members of the household is more than the proportional income they should contribute to the household

If we instead evaluate the proportion of households whose members contributed at least the proportional income that would preserve them from becoming a below median-income household, regardless of the income of the household head, we find 31% of EU-EFTA-headed families did, share that drops to 27% for the Swiss and 23.2% for the non-EU/EFTA-headed families. These results would confirm, at least in an initial stage of the family reunion, that the contribution of non-EU/EFTA family members is lower than that of EU/EFTA migrants (H1). This difference should be interpreted in relation to the greater ease found by members of EU/EFTA families to enter the Swiss labour market but also in connection to the different reasons for migration and initial attitudes towards labour market participation of the different groups.

On the basis of the shortage in family and institutional support for childcare and the high cost of childcare in Switzerland, our second hypothesis (H2) proposed that the contribution of family members in reunions involving children would be less than those of just partners. As expected, the proportion of families whose members contributed the proportional income necessary to cover their weight over the household median threshold is lower among families with children. Nevertheless, their monetary contribution was higher and as noted above, that contribution had a greater impact stabilizing the economic situation of those households, in particular among families with 2 or 3 children.

### Results 1.3: the Hybrid Nature of Swiss-Headed Reunited Families

As expected, the financial position of the heads in 2012 and the contribution of family members in 2014 to the family budget are both powerful predictors of the economic situation of the household once the reunion has been carried out. To test how these set of variables impact the likelihood of living below the median income after the reunion for the different population groups, we fit a logistic regression model including all households whose heads were living above the median income in 2012 (5237 cases). The contribution of family members to the family budget is modelled as an ordinal variable in relative terms using the following intervals: no contribution, less than 20%, between 20 and 40%, 40 and 60% and more than 60% of the family budget. The model also controls for other sociodemographic characteristics of the population available in our dataset (region of birth of the head and its age, sex and income quartile and the region of birth of the reunited partner) and the type of reunion. We exclude the reunion of children without a partner given the few cases included in the dataset.

The results of the models (Table 5) corroborate certain aspects that were already present in the transition analysis. For example, a higher level of income of the head in 2012 is negatively associated with the probability of moving below the median income after the reunion. Regarding the type of reunion, although the reunion of a partner and children carries a higher risk of not reaching the median income compared to the reunion of only the couple, the risk is greater for the reunion of a partner and a child. This difference can be explained in relation to the age of the children in these two types of reunions (in reunions with only one child, the child is on average younger) and the possibility their mothers of joining the labour market. The model also highlights the lower risk of households headed by a woman (19% of this sample with 1002 cases) of falling below the median income after reunion, which would be related to the fact that men arriving for family reasons face fewer difficulties to incorporate into the labour market than women. Regarding the contribution of family members, it reduces the risk of not reaching the median income when it does not exceed 60% of household income. When it does, it most commonly goes along with a poor economic performance of the head and it is connected with a higher risk of living in VI or VLI households.

**Table 5:** Logistic models, outcome variable: living in a low or very low-income household

Model 1					
Coefficients					
	Estimate	Std. error	*z* value	Pr( >|*z*|)	
(Intercept)	− 1.685E + 00	1.023E − 01	− 16.465	< 2e − 16	***
Region of birth of head (ref. EU/EFTA)					
Non-EU/EFTA	7.506E − 01	8.295E − 02	9.049	< 2e − 16	***
Switzerland	− 2.342E − 01	9.472E − 02	− 2.473	1.34E − 02	*
Sex (ref. Men)					
Women	− 4.014E − 01	1.012E − 01	− 3.965	7.33E − 05	***
Age	1.101E − 04	4.57E − 05	2.41	0.0159	*
Signif. Codes:	0 ‘***’	0.001 ‘**’	0.01 ‘*’	0.05 ‘.’	
Model 2 with control variables					
Coefficients					
	Estimate	Std. error	*z* value	Pr( >|*z*|)	
(Intercept)	− 7.402E − 01	1.513E − 01	− 4.893	9.94E − 07	***
Region of birth of head (ref. EU/EFTA)					
Non-EU/EFTA	5.184E − 01	1.218E − 01	4.257	2.07E − 05	***
Switzerland	1.442E − 01	1.278E − 01	1.129	2.591E − 01	
Sex (ref. Men)					
Women	− 3.506E − 01	1.251E − 01	− 2.803	0.00506	**
Age	2.611E − 04	5.625E − 05	4.641	3.47E − 06	***
Income quartile head (ref. q1)					
q2	− 1.074E + 00	1.001E − 01	− 10.726	< 2e − 16	***
q3	− 2.474E + 00	1.311E − 01	− 18.872	< 2e − 16	***
q4	− 3.521E + 00	1.773E − 01	− 19.856	< 2e − 16	***
Region of birth of partner (ref. EU/EFTA)					
Non-EU/EFTA	2.656E − 01	1.140E − 01	2.33	0.0198	*
Income contribution family members (ref. NO CONTR.)					
Less 20%	− 6.146E − 01	1.118E − 01	− 5.498	3.85E − 08	***
20–40%	− 1.874E + 00	1.574E − 01	− 11.903	< 2e − 16	***
40–60%	− 1.609E + 00	1.674E − 01	− 9.609	< 2e − 16	***
More than 60%	4.735E − 01	2.077E − 01	2.28	0.02263	*
Type of reunion (ref. Partner)					
Partner + 1 child	9.058E − 01	1.064E − 01	8.512	< 2e − 16	***
Partner + 2 or more children	1.097E + 00	1.29E − 01	8.507	< 2e − 16	***
Signif. Codes:	0 ‘***’	0.001 ‘**’	0.01 ‘*’	0.05 ‘.’	

Source: Own elaboration with data from the Population Registers and the Old Age and Survivors Insurance

The most relevant aspect shown by the results refers to the situation of the three population groups in relation to the risk of falling below the median income once all control variables are included in the model. While the observed values trace a scale from the lowest risk of Swiss-headed households of becoming LI or VLI after the reunion to that of those headed by a non-EU/EFTA country citizen, once controls are included, the risk for Swiss-headed households increases till exceeding that of EU/EFTA households, at the same time as the differences with the other two groups disappear. The variation in the coefficients can be explained by the higher share of reunions involving children among EU/EFTA households, the high prevalence of non-EU/EFTA partners (and children) in the Swiss-headed households and the different share of contributors to the family income between groups, which is 20% for EU/EFTA households, 15% for Swiss-headed households and 13% for the non-EU/EFTA. This change highlights similarities between Swiss and non-EU/EFTA reunited households. On the one hand, it is possible that the convergence is connected with a more traditional view of gender roles and women’s participation in the labour market, on the other, due to the shared difficulties, namely, the lack of recognition of qualifications and command of the local language that the reunited family members from non-EU/EFTA countries encounter to participate in the Swiss labour market.

### Results 1.4: Family Reunion and Income 5 Years Later

**Fig. 2:**
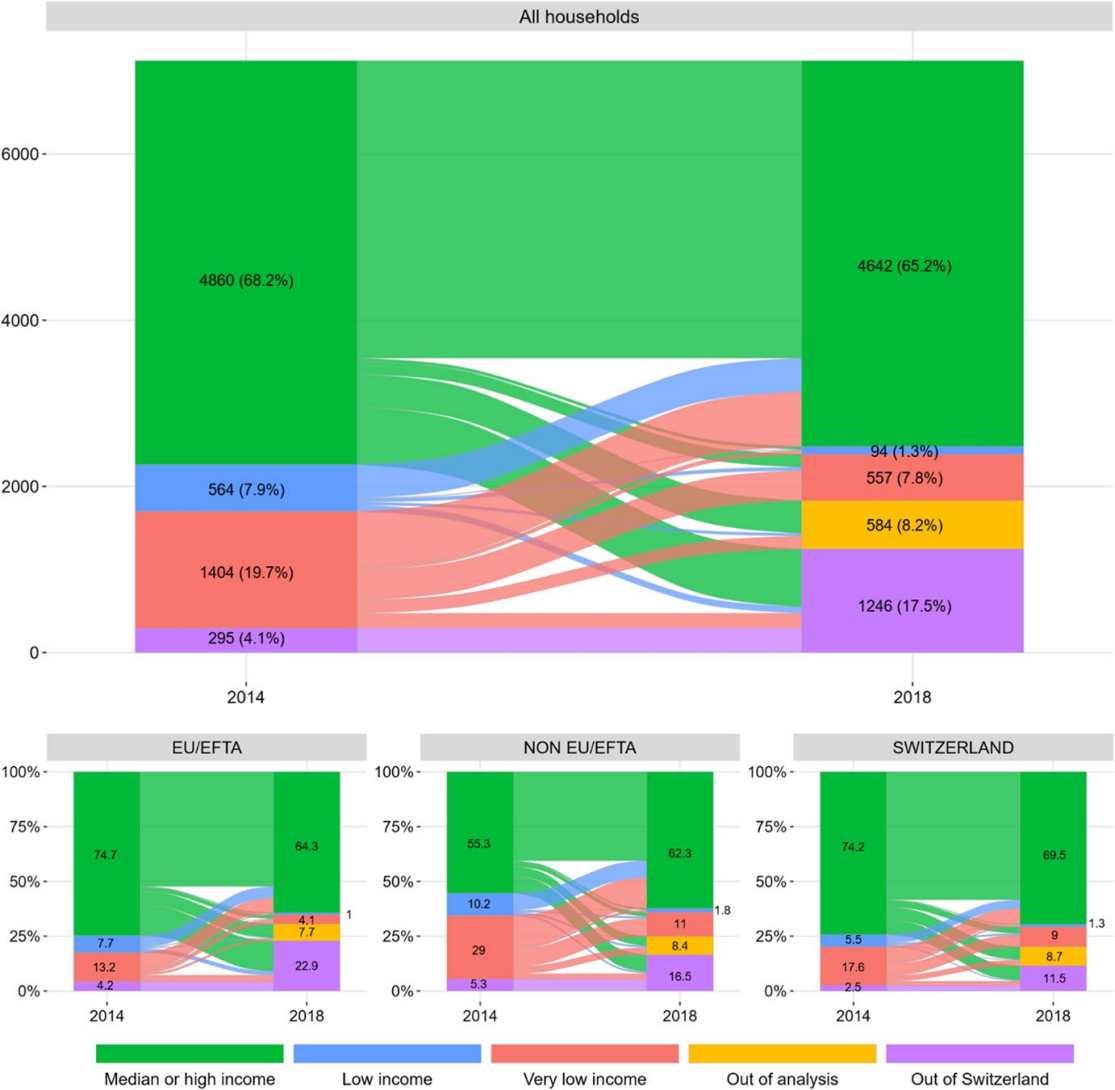
Transition between income states of households in 2013 and 2018. Source: Own elaboration with data from the Population Registers and the Old Age and Survivors Insurance

The last analytical step of this paper consists of reassessing the economic situation of households 5 years after the reunion. The changes occurred during the 5 years force us to introduce one new category as possible states: ‘Out of the analysis’ category where we include those households that turned to be single-person households (7.6% of our original sample) and those composed by 6 or more people (1% of the original sample) in 2018 (Fig. 2).

Five years after the reunion, we find in Switzerland 5997 of the initial heads, which accounts for 84.2% of our original sample (7,123 cases). These households add up to a total of 17,662 people in 2018. Regarding families that are Out of Switzerland in 2018, data shows that they were mostly MHI households in 2014 (79%), headed by an EU/EFTA citizen (52%) and with a majority of heads (40%) in the fourth income quartile (more than 97,808 francs per year). Therefore, leaving Switzerland does not seem to be connected, in the case of family reunion, to the economic failure of the migration project. Among those families in LI or VLI households in 2018 (651 cases, 9.1%), the first thing to note is that 7 out of 10 were in this situation in 2014. The persistence of this disadvantage is mainly related to the interaction between the lack of sufficient contribution of income by family members to the family budget and its growth in size between 2013 and 2018, so it is not surprising that most of them are non-EU/EFTA-headed families.

In 2018, two-thirds of reunited families (4642 cases) were living in MHI households. This group is composed of those who were already in this situation in 2014 (3579 cases accounting for 77.1% of the total families above median income in 2014) plus 1063 families who were living in LI (404) or VLI (659) households back in 2014. In relative terms, this means 23% of MHI households in 2018 were LI or VLI households in 2014, share that ranges between 16% for the Swiss-headed families (230 cases) and 34.8% (385 cases) for the non-EU/EFTA ones. Hence, the last questions we want to address are, how can we explain the change of state between the two years? Was it a consequence of the increase in the income of the head of the family, her or his partner and/or children reaching labour age or a combination of both factors? And for how many families living in MHI households in 2018 was the contribution of the rest of the household members crucial to finding themselves in such situation? To address these questions, we first reassess the proportion of households whose members contributed, at least, their proportional median income to the household budget in 2018. Then we subtract the contribution of family members from the household income and reassess their positions in relation to the median income by households’ size in 2018.

After 4 years in Switzerland, the proportion of households whose members contributed, at least, their proportional income to the household budget raised from 13.5 to 29% for non-EU/EFTA-headed households and from 21.3 and 15 to 37% for the EU/EFTA and Swiss-headed households respectively. The greater growth in contributions to the family budget of members of reunited families headed by a Swiss citizen compared to those headed by a non-EU/EFTA national should be interpreted both in relation to the preponderance of the reunion of partners and the lower presence of children in these households, but also as a consequence of the access to a broader social network and an easier assimilation of the language that benefit the reunited members of these households and have a positive impact on their professional integration. When the contribution of reunited members is subtracted from the household income, we find that for half of those LI or VLI households headed by a Swiss or an EU/ETFA national, and for two-thirds of households headed by a non-EU/EFTA one, their contribution was crucial to find them living in MHI households 2018. In the case of those who lived in MHI households in 2014 and 2018, the contribution of family members prevented 13% of households headed by a Swiss, 15% of EU/ETFA and 29% of those headed by a non-EU/EFTA person from living below the median income in 2018. The importance of this contribution becomes more significant considering the increase in size (and consequently median income threshold) experienced by most of these households between 2013 and 2018.

In summary, 5 years after the reunion, and despite the differences in the initial economic position of the reunited families, data on household income indicates that there has been a process of convergence between the three groups regarding the proportion that achieves to live in MHI households. This process is related, on the one hand, to the different survival rate of families reunited by region of birth of their heads, but, on the other, to the increase in the number of relatives who join the labour market and contribute the necessary income to cover, at least, the proportional part of the rise in the income threshold caused by their incorporation. The notable increase among Swiss-headed families of this proportion (from 15% in 2013 to 37% in 2018) leads to a convergence with those of the EU-EFTA and highlights once again the hybrid nature of reunited families headed by a Swiss citizen.

## Conclusion and Discussion

Family migration has gained prominence as a reason for international mobility in both Switzerland and other European countries. However, research aimed at evaluating the economic performance of reunited families has been constrained by the unavailability of individual income data and/or household composition, among others. The joint use of population registers with information on the income of the population residing in Switzerland has allowed us to overcome this limitation and to assess the economic performance of reunited families.

The growing importance of family reunification has fuelled public and political debates about its scope and convenience. These debates in Switzerland have been mainly related to the fear that family reunion could lead to the need for social assistance and its potential costs to the welfare state. The analysis carried out here has examined the relationship between family reunion and income from a family (household) perspective, trying to overcome the limitations of the individualistic neo-classic perspective from which this topic has most frequently been addressed. The results obtained present a series of characteristics of family reunion in Switzerland and its connection with the potential transit to a disadvantaged income position of reunited families. These results, in turn, may contribute to empirically informing those debates and should be considered in the design of public policies in this area.

Family reunification in Switzerland is a process driven by men (three out of four of those who initiate the process) and more frequently oriented towards the reunion of the partner than the reunion of a partner and children. Regarding the relation between family reunification and income, the results clearly show that the great majority of reunited households achieve acceptable financial standards that allow them to live in MHI households. They also reflect that among households living below the median income the year of the reunion, such situation is connected, for half of them, with a poor economic performance of the head before the reunion. This shows, in time, that even in a country with a highly restrictive immigration policy such as Switzerland, family reunion may occur before the head of the household achieves economic, housing and/or labour stability in the country.

The results of the logistic model show that even after controlling for the economic position of the head and by a number of available sociodemographic characteristics of individuals and households, differences between groups in relation to the likelihood of falling below the median income after the reunion remain. Families headed by non-EU/EFTA migrants have a greater chance of living below median income after family reunion, which indicates the presence of other structural disadvantages that we cannot observe with the available data but that have been addressed in other studies (Auer & Fossati, 2019; Riaño, 2021; Wanner, 2019; Zschirnt & Ruedin, 2016). Furthermore, given that a majority of migrants from EU/EFTA countries come from bordering countries with similar languages, one main obstacle for professional integration is removed for this group. Before controlling for the income level of the head previous to the reunion and the contribution of family members to the household income, Swiss-headed households resemble those with an EU-EFTA head in relation to their economic performance. However, once controls are included in the model, they appear closer to the non-EU/EFTA-headed ones. This change is basically explained by the differences in the origin of the reunited partner, with Swiss and non-EU heads having been more likely to reunite with non-EU partners (who face higher limitations to enter the labour market). One major limitation of this research is the unavailability of information on the level of education of the reunited family members. This prevents us from weighing the results related to the economic contribution of family members to the household budget in relation to labour overqualification, which generally affects the migrant population and with greater intensity migrants from non-EU/EFTA countries living in Switzerland (see Pecoraro & Wanner, 2019; Gerber & Wanner, 2019).

In addition to the place of birth of the head, sex is also a strong driver of professional integration and therefore contributions to household income. Families headed by women exhibit a lower likelihood of living in VI or VLI households after family reunion than those headed by men. This can be explained, first, by the fact that women arriving for family reasons encounter more difficulty integrating into the host labour market than men and, second, by the gender gap in salaries. This result is also in line with the literature in Switzerland; migrant men are less likely to be overeducated for the jobs they obtain than are the women (Pecoraro, 2005; Pecoraro & Wanner, 2019, 2019; Riaño & Baghdadi, 2007; Wanner, 2019) and show a lower probability of being unemployed (Wanner, 2019). Family configuration, i.e. bringing a partner with or without children, also has a significant impact on the relation of reunited families with income. Reunification with children increases the likelihood of moving below the median income. This result might be specifically related to the Swiss context; the observed shortage in family and institutional support for childcare and the high cost of childcare (see Adema et al., 2014; Felfe et al., 2013) may impede parents, especially women, from economically contributing to household income due to the care burden.

The nature of the data allows us to examine the situation of reunited families 5 years after the year of reunification. We found that more than half of families living in LI households and one-third of those in VLI households in 2014 had overcome that situation by 2018. Overall, after 5 years, only a limited number of families live in LI or VLI households. This is consistent with the well documented trend within migration studies connecting the length of residency in the host country with a positive professional integration, and therefore with better economic prospects for the entire family. Data also reflects that for half of these families, the contribution of household members was crucial and prevented them from living in economically disadvantaged households in 2018. In the case of family reunion, (re)emigration does not seem to be connected to the economic failure of the migration project as postulated by the neoclassical theories (Constant & Massey, 2003) but to the higher international mobility of skilled migrants.

From the right for family reunion, across the professional integration of secondary migrants, the economic success of families is mainly driven by the intersections of nationality, gender, socioeconomic status, family configuration and family transitions, which creates, for some of them, long-term income disadvantages. Analysing work-family trajectories under an intersectional approach (see, e.g. Riaño, 2021; Aisenbrey & Fasang, 2018) contributes to identifying the cumulative disadvantages faced by reunited families composed of a primary migrant with poor economic performance and his or her family members. Professional integration of family migrants may take time in particular for the non-EU/EFTA population, making the financial situation of families tenuous in the interim. However, and despite the differences in the initial income position of the reunited families, the results presented indicate a process of convergence between the different groups in relation to achieve living in MHI households 5 years after the reunion. This process is particularly well illustrated by the hybrid nature of reunited families headed by a Swiss citizen, which initially resemble those headed by a non-EU/EFTA person but end up behaving similar, in terms of the contribution their members make to the household budget, to EU/EFTA-headed families.

## Footnotes

1 https://www.parlament.ch/fr/ratsbetrieb/suche-curia-vista/geschaeft?AffairId=20190464, consulted on 03.06.2022.

2 The evaluation of the accommodation is left up to cantonal authorities and is highly discretionary, since an accommodation is deemed suitable if it reflects the typical living conditions of Swiss citizens (SEM, 2021).

3 According to Cooke (2013:818), ‘A tied migrant is usually defined as an individual whose family migrated but who would not have chosen to move if single […]’.

4 OASI is the English translation for AVS (*Assurance-Vieillesse et Survivants*) in French and for AHV *(Alters- und Hinterlassenenversicherung*) in German.

5 The ‘head’ of a household can be foreign-born (family reunification) or Swiss-born (family formation and/or reunification).
